# P-678. Respiratory Syncytial Virus infection and bacterial colonization in children under 24 months

**DOI:** 10.1093/ofid/ofaf695.891

**Published:** 2026-01-11

**Authors:** Patricio L Acosta, Agustina Denardi, Noelia Iraizos, Ana Fernandez, F Martín Ferolla, Maria Marta Contrini, Eduardo Lopez

**Affiliations:** Pediatric Infectious Disease Program, Hospital de Niños Ricardo Gutiérrez, Universidad de Buenos Aires, CONICET, Buenos Aires, Ciudad Autonoma de Buenos Aires, Argentina; Hospital de Niños "Dr. Ricardo Gutierrez", CABA, Ciudad Autonoma de Buenos Aires, Argentina; Hospital de Niños Ricardo Gutierrez, Ciudad Autónoma de Buenos Aires, Buenos Aires, Argentina; Hospital de Niños Ricardo Gutierrez, Ciudad Autónoma de Buenos Aires, Buenos Aires, Argentina; Hospital de Niños Ricardo Gutierrez, Ciudad Autónoma de Buenos Aires, Buenos Aires, Argentina; Hospital de Niños Ricardo Gutiérrez, CABA, Ciudad Autonoma de Buenos Aires, Argentina; Hospital de Niños Ricardo Gutierrez, Ciudad Autónoma de Buenos Aires, Buenos Aires, Argentina

## Abstract

**Background:**

Respiratory syncytial virus (RSV) is a major cause of acute lower respiratory infection (ALRI) in young children. Annually, RSV results in 2.8–4.3 million hospitalizations and up to 199,000 deaths, primarily in developing countries. The mechanisms underlying the wide range of RSV disease severity are likely multifactorial but remain poorly understood. Previous studies suggest that colonization of the upper respiratory tract by certain bacteria—such as *Streptococcus pneumoniae* (Spn), *Haemophilus influenzae* (Hi), and *Moraxella catarrhalis* (Mrx)—may influence host immune responses and affect clinical outcomes.

**Figure:**
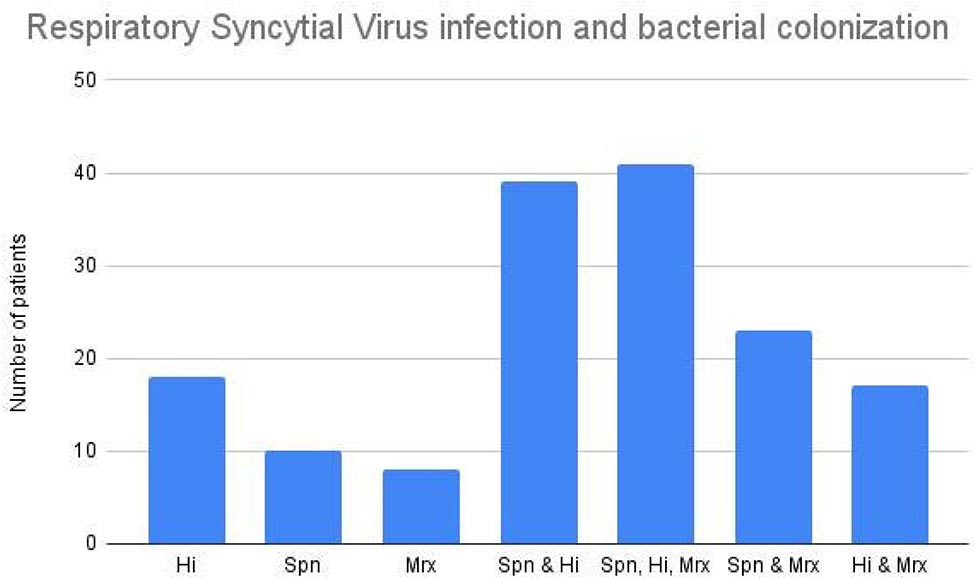

**Methods:**

A prospective cohort study was conducted at Hospital de Niños Ricardo Gutiérrez, Buenos Aires, during the 2019 RSV season. Children under 24 months hospitalized with RSV infection confirmed by qPCR were included. Patients were categorized by clinical severity: mild (no supplemental oxygen required), severe (supplemental oxygen required), and life-threatening disease (ICU admission needed). Nasal aspirates were collected, and qPCR was performed to detect Spn, Hi, and Mrx.

**Results:**

A total of 173 patients were included (55% male). Of these, 15.6% had mild disease, 75.7% severe disease, and 8.7% life-threatening disease (LTD). Median age was 5 months (IQR 1–24). Bacterial colonization was identified in 156 patients (figure 1).Hospitalization median length of stay was 4 days (IQR 3–7) in colonized vs. 4 days (IQR 3–8) in non-colonized patients. Antibiotic treatment duration showed no significant difference: 5 days (IQR 2–9) in colonized vs. 6 days (IQR 4–9) in non-colonized. Mrx colonization was associated with a lower rate of life-threatening disease (p = 0.018). No deaths occurred.

**Conclusion:**

In hospitalized children with RSV infection, upper respiratory bacterial colonization was not associated with increased clinical severity, length of stay, or antibiotic duration. Mrx colonization may be linked to a reduced risk of life-threatening disease.

**Disclosures:**

All Authors: No reported disclosures

